# The Assessment of Auditory Function in High-Risk Neonates and Infants Using Brainstem-Evoked Response Audiometry (BERA)

**DOI:** 10.7759/cureus.66666

**Published:** 2024-08-12

**Authors:** Rashmi P Rajashekhar, Sunanda Devi Putta, Aishwarya Kothari, Mayur Ingale

**Affiliations:** 1 Otolaryngology-Head and Neck Surgery, Dr. D. Y. Patil Medical College, Hospital and Research Centre, Dr. D. Y. Patil Vidyapeeth (Deemed to be University), Pune, IND

**Keywords:** high-risk infants, high-risk neonates, auditory brainstem response (abr), auditory functions, bera

## Abstract

Introduction

Hearing impairment in neonates and infants is a critical concern due to its potential to impede language acquisition, cognitive development, and overall quality of life. Brainstem-evoked response audiometry (BERA) stands out as a valuable diagnostic tool. The early detection of hearing impairments is paramount in neonatal care. Hearing loss during infancy can impede speech and language development, social interaction, and academic achievement. High-risk neonates, including those born prematurely or with low birth weight, have a heightened susceptibility to hearing impairment due to various factors such as exposure to ototoxic medications, mechanical ventilation, and complications associated with prematurity.

Methods

A hospital-based prospective study was conducted in the department of otorhinolaryngology; the study focused on high-risk neonates and infants from the outpatient department and inpatient department. The study was conducted from October 2022 to March 2024. A sample size of 70 patients was taken, including high-risk neonates and infants. Healthy term neonates and healthy infants were excluded from the study.

Results

In the current study, there were 40 males and 30 females. Among the infants surveyed, prematurity was the most prevalent risk factor, followed by perinatal asphyxia. Low birth weight was observed in 43% of cases, while hyperbilirubinemia and neonatal sepsis were the next. Among the 70 infants assessed, 50% were found to have normal hearing. Mild hearing loss was observed in 23% of cases, while 14% had moderate hearing loss. Severe and profound hearing loss were less common.

Conclusion

Our study highlighted the importance of early and routine auditory screening using BERA in high-risk neonates and infants, revealing a significant prevalence of hearing loss linked to various risk factors such as premature babies, low birth weight, hyperbilirubinemia, neonatal intensive care unit stay, perinatal asphyxia, and ototoxic drugs during pregnancy. Prematurity is the most common risk factor. For language development, early diagnosis and intervention were crucial. If babies have profound sensorineural hearing loss, they can go for a cochlear implant.

## Introduction

Hearing impairment in neonates and infants is a critical concern due to its potential to impede language acquisition, cognitive development, and overall quality of life. At the same time, congenital hearing loss affects approximately 1-3 per 1000 live births globally and 1-8 per 1000 births nationally. The prevalence is notably higher in high-risk neonates, including those born prematurely (34-36 weeks), with low birth weights ranging from 1500 g to 2400 g, and with a family history of hearing loss [1].

This technique offers several advantages, particularly in high-risk populations, including its noninvasiveness, reliability, and ability to assess infants regardless of their developmental stage or level of cooperation [2].

The application of brainstem-evoked response audiometry (BERA) to high-risk neonates and infants presents a unique set of challenges and opportunities. Understanding the underlying principles of brainstem-evoked response audiometry, its clinical utility, and the specific considerations in this population is essential for optimizing its effectiveness in early hearing assessment [3].

The early detection of hearing impairments is paramount in neonatal care. Hearing loss during infancy can impede speech and language development, social interaction, and academic achievement [4]. High-risk neonates, including those born prematurely or with low birth weight, have a heightened susceptibility to hearing impairment due to various factors such as exposure to ototoxic medications, mechanical ventilation, and complications associated with prematurity. Moreover, genetic predispositions and family history further elevate the risk of congenital hearing loss in this population [4].

Studies explore various aspects, including the sensitivity and specificity of BERA in detecting hearing impairment, the impact of prematurity and low birth weight on auditory function, and the effectiveness of early intervention programs in improving developmental outcomes [5].

## Materials and methods

A hospital-based observational study was conducted at the department of otorhinolaryngology in a tertiary care hospital in India from October 2022 to March 2024. The study focused on high-risk neonates and infants from the ENT and pediatric outpatient and inpatient departments.

Sample size

Considering the incidence of hearing loss in high-risk babies at 70%, with 95% confidence and an accuracy of 11%, the minimum sample size required was calculated to be 67. However, this number was rounded up to 70.

Inclusion criteria comprised high-risk neonates and infants of age group below one year, and high risk includes prematurity, low birth weight, hyperbilirubinemia, neonatal sepsis, perinatal asphyxia, and ototoxic drugs, while exclusion criteria comprised healthy term neonates and infants. A total of 70 high-risk babies were evaluated using brainstem-evoked response audiometry.

Details such as demographic and clinical characteristics of the sample, including gestational age, sex, birth weight, and identified risk factors for hearing impairment, were collected, and the infants were assessed using BERA.

Statistical analysis

Data collected was entered into Microsoft Excel 365 (Microsoft Corp., Redmond, WA), and statistical analysis was conducted to assess the prevalence of hearing loss among the high-risk neonates and infants who participated in the study. Descriptive statistics were used to summarize the details collected. The software used for analysis was jamovi v2.4 (The jamovi project, Sydney, Australia).

Ethics approval

Approval was obtained from the Institutional Ethics Sub-Committee of the Dr. D. Y. Patil Medical College, Hospital and Research Centre (vide reference number IESC/PGS/2022/122), before the start of the study. Informed consent from the parents was obtained before obtaining the participant's details and assessing using BERA.

## Results

The present study assessed 70 high-risk infants and neonates. There were 40 male infants, making up 57% of the group, while 30 female infants accounted for the remaining 43%.

The median birth weight is 2500 g, with an interquartile range of 500 g. The mean gestational age was 35 weeks, with a standard deviation of 2.5 weeks. This indicates that, on average, the gestational age was around 35 weeks, with most individuals falling within approximately 2.5 weeks of this average.

Table 1 summarizes the risk factors among the assessed infants. Prematurity (41, 59%) was the most prevalent risk factor, followed by low birth weight (30, 43%).

**Table 1:** Distribution of risk factors among infants Some infants had multiple risk factors such as low APGAR score, prolonged ventilation, meningitis, and congenital intrauterine anomaly APGAR: appearance, pulse, grimace, activity, and respiration

Risk Factor	Number of Infants (N=70)	Percentage (%)
Prematurity	41	59
Low Birth Weight (<2500 g)	30	43
Hyperbilirubinemia	16	23
Neonatal Sepsis	10	14
Perinatal Asphyxia	22	31
Ototoxic Medication	10	14

Table 2 summarizes the hearing status of the infants. Half of the infants, 35 in total, had normal hearing, and the rest of the infants (35) had hearing loss. Severe hearing loss was present in four infants, making up 6%, and profound hearing loss was noted in five infants, comprising 7% of the total.

**Table 2:** BERA results of the infants (hearing status) BERA: brainstem-evoked response audiometry

Hearing Status	Number of Infants (N=70)	Percentage (%)
Normal Hearing	35	50
Mild Hearing Loss	16	23
Moderate Hearing Loss	10	14
Severe Hearing Loss	4	6
Profound Hearing Loss	5	7

## Discussion

This hospital-based observational study was conducted between October 2022 and March 2024 to assess auditory function in high-risk neonates and infants using BERA. The study found that 35 (50%) high-risk infants exhibited abnormalities in BERA. Comparatively, Champion included a significant proportion of preterm infants (44%) and those with low birth weight (46%), while our study had a higher proportion of prematurity (59%) but a slightly lower proportion of low birth weight (43%). Both studies noted male predominance in high-risk groups. Champion reported that of 50 high-risk infants, 44 (88%) had impaired BERA, and six (12%) had normal-threshold hearing [6].

Champion observed a higher prevalence of hearing loss in infants with multiple risk factors (36/50 versus 8/50). Both studies identified low birth weight and hyperbilirubinemia as significant risk factors. Champion found strong correlations between these factors and hearing loss. The present study noted low birth weight (43%) and hyperbilirubinemia (23%) as prevalent but did not provide specific statistical measures for their correlation with hearing loss due to limited sample size. Both studies recognized the impact of consanguinity and family history of hearing loss, though Champion highlighted a higher percentage of infants from consanguineous marriages (36%) compared to our broader categorization [6].

Champion provided a breakdown of hearing impairment severity but did not detail percentages across categories (mild, moderate, severe, and profound). The present study adds details for mild (23%), moderate (14%), severe (6%), and profound (7%) hearing loss [6].

The varying prevalence of hearing loss by risk factor highlights the need for multidisciplinary approaches in managing these infants. For example, infants with perinatal asphyxia or neonatal sepsis may benefit from additional neurological assessments, and interventions include mental status evaluation, cranial nerve examination, cerebellar functioning, and reflexes to address potential central auditory processing disorders. The follow-up results underscore the importance of ongoing monitoring and intervention, as auditory function can change over time, necessitating adjustments in management strategies [7-9].

Thirunavukarasu et al. addressed the importance of early detection and intervention [10]. In their study, they screened 125 infants and children, of whom 44 (35.2%) had sensorineural hearing loss. Among those, 21 were males, and 23 were females. The most common risk factor included consanguineous marriage [10].

Warasanti et al., in their study, had 19 high-risk infants; most of them were below the age of one month, and 10 infants, almost half, had positive BERA results. Asphyxia was found to be the most common risk factor [11].

Sharma et al. had 45 high-risk infants in which severe-to-profound sensorineural hearing loss was seen in five infants, and with the distribution of risk factors, severe hearing impairment is with neonatal hyperbilirubinemia, and profound hearing impairment is with low birth weight [12].

However, differences in population characteristics and identified risk factors highlight the need for targeted screening and preventive strategies tailored to specific high-risk groups.

The correlation of birth weight and gestational age with hearing loss emphasizes the vulnerability of preterm and low-birth-weight infants to auditory impairments. These findings support the implementation of universal hearing screening programs in neonatal intensive care units and follow-up clinics. Gender differences in hearing loss prevalence suggest that male infants may require closer monitoring and possibly different screening protocols to ensure early detection and intervention [13].

This study addresses the significant need for the early detection of hearing impairments in high-risk neonates and infants, a population particularly vulnerable to auditory dysfunction due to factors such as prematurity, low birth weight, hyperbilirubinemia, and neonatal infections. The early identification and management of hearing impairments are vital for preventing delays in speech, language, cognitive, and social development, ultimately improving the quality of life and developmental outcomes for these high-risk infants.

Study limitations

Sample Size

Although the sample size of 70 is based on statistical calculations, it remains relatively small, which may limit the generalizability of the findings. Larger studies are needed to confirm these results and to enhance their applicability to broader populations.

Single-Center Study

Conducted in a single hospital setting, the study's findings may not be representative of other institutions or regions. Differences in healthcare practices and population demographics could influence the prevalence and characteristics of hearing loss.

## Conclusions

This comparative study evaluated the outcomes of hearing loss in patients with risk factors. Our results demonstrate that infants and neonates below one year who had risk factors such as prematurity, low birth weight, hyperbilirubinemia, neonatal sepsis, perinatal asphyxia, and ototoxic medications have hearing loss. Significant risk factors in our study include prematurity, which includes preterm from 34 to 36 weeks, and low birth weight (1500-2400 g). Universal auditory screening is important in high-risk populations and neonatal intensive care units to optimize auditory and developmental outcomes. The early identification and management of hearing impairments can significantly improve language, cognitive, and social development outcomes. Prematurity is the most common risk factor, with a higher prevalence of hearing loss. High-risk and neonatal intensive care unit-stayed babies were taken into consideration in our study. In an age of less than two years, hearing improvement can be seen, but after five years, it is irreversible. In such cases, early detection is required by undergoing BERA so that if babies have profound sensorineural hearing loss, they can go for a cochlear implant. In deaf-mutism babies, hearing can be reversible if early detection is done.
